# Supplementary material for: The Evolution and Characterization of the RNA Interference Pathways in Lophotrochozoa
Source: Genome Biol Evol. 2024 May 7;16(5):evae098. doi: 10.1093/gbe/evae098 (PMC11114477; doi:10.1093/gbe/evae098)
Supplement: evae098_Supplementary_Data [file evae098_supplementary_data.zip › Supplementary_materials.zip!/selected_miRNAs/NoGe_selected_miRNAs/mirdeep_pdf_files/NMRB01000261.1_Notospermus_geniculatus_strain_Ushimado_scaffold261,_whole_genome_shotgun_sequence_8544.pdf]

Provisional ID : NMRB01000261.1_Notospermus_geniculatus_strain_Ushimado_scaffold261,_whole_genome_shotgun_sequence_8544
Score total : 155540.1
Score for star read(s) : 3.9
Score for read counts : 155531.5
Score for mfe : 1.8
Score for randfold : 0
Score for cons. seed : 3
Total read count : 305080
Mature read count : 303553
Loop read count : 0
Star read count : 1527

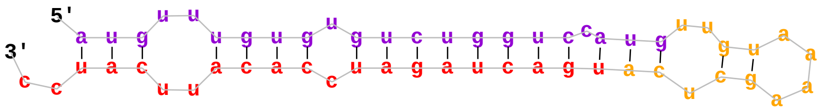

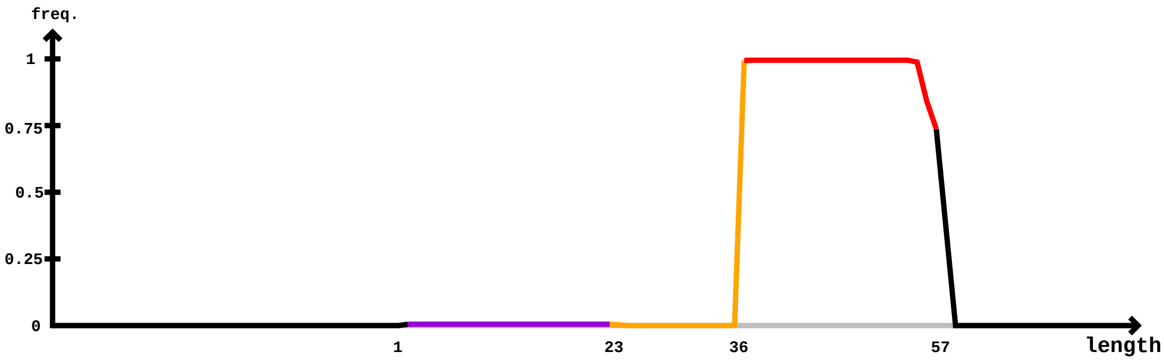

Star **Mature**

5'- cauucaacauugacagugcagugccagucuuuaggauguuugugugucugguccauguuguaaaagcucaugacuagauccacauucauccugacggcuuggaaucaucua -3'	obs		
cauucaacauugacagugcagugccagucuuuaggauguuugugugucugguccauguuguaaaagcucaugacuagauccacauucauccugacggcuuggaaucaucua	exp		
.......((((......)))))(((((.((((((((..((((.((((((((.(((..((....)).)))))))))))).)))))..)))))))).))).))).........	reads	mm	sample
...................................auguuugugugucugguGc...	1	1	Mm3
...................................auguuugugugucugguccaug..	61	0	Mm3
...................................auguuugugugucugguAcaug..	1	1	Mm3
...................................auguuugugugucugguGcaug..	1	1	Mm3
...................................auguuugugugucugguccaugu...	2	0	Mm3
...................................auguuugugugucugguccauguugu..	1	0	Mm3
....augacuagauccacaCucauc.....................	2	1	Mm3
....augacuagauccacaCucaucc....................	8	1	Mm3
....ugacuagauccacaCuca.......................	30	1	Mm3
....ugacuagauccacaCucau......................	235	1	Mm3
....ugacuagauccacaCucauc.....................	3336	1	Mm3
....ugacuagauccacauucauU.....................	1	1	Mm3
....ugacuagauccacaCucaucc....................	31930	1	Mm3
....ugacuagauccacauucaucc....................	9	0	Mm3
....ugacuagauccacaGucaucc....................	3	1	Mm3
....ugacuagauccacaAucaucc....................	1	1	Mm3
....ugacuagauccacauucauccu...................	2	0	Mm3
....ugacuagauccacaCucauccu...................	2807	1	Mm3
....gacuagauccacaCucau......................	1	1	Mm3
....gacuagauccacaCucauc.....................	6	1	Mm3
....gacuagauccacaAucaucc....................	1	1	Mm3
....gacuagauccacaCucaucc....................	35	1	Mm3
....gacuagauccacaCucauccu...................	5	1	Mm3
....cuagauccacaCucaucc....................	1	1	Mm3
...................................auguuugugugucugguGca..	1	1	Fm3
...................................auguuugugugucuggucca..	9	0	Fm3
...................................auguuugugugucugguccau...	6	0	Fm3
...................................auguuuguguAucugguccaug..	4	1	Fm3
...................................auguuugugugucugguUcaug..	4	1	Fm3
...................................auguuugugugucugguAcaug..	7	1	Fm3
...................................auguuugugugucuggGccaug..	2	1	Fm3
...................................auguuugugugucuggAccaug..	1	1	Fm3

Star **Mature**

```
cauucaacauugacagugcagugccagucuuuaggauguuugugugucugguccauguuguaaaagcucaugacuagauccacauucauccugacggcuuggaaucaucua
...................................auguuugugugucuggCccaug......................................................   3        1   Fm3
...................................auguuugugugucugguccaug......................................................   555      0   Fm3
...................................auguuuguguUucugguccaug......................................................   1        1   Fm3
...................................auguuugugugucugAuccaug......................................................   1        1   Fm3
...................................auguuugugugucugguGcaug......................................................   1        1   Fm3
...................................auguuugugugucugguccauA......................................................   1        1   Fm3
...................................auguuuguUugucugguccaug......................................................   1        1   Fm3
...................................auguuugugugucugguccaugA.....................................................   2        1   Fm3
...................................auguuugugugucugguccaugu.....................................................   1        0   Fm3
...................................auguuugugugucugguccauguu....................................................   2        0   Fm3
...................................auguuugugugucugguccauguug...................................................   2        0   Fm3
...................................auguuugugugucugguccauguugu..................................................   1        0   Fm3
....................................uguuugugugucugguccaug......................................................   10       0   Fm3
....................................uguuugugugucuggucUaug......................................................   1        1   Fm3
....................................uguuugugugucuCguccaug......................................................   6        1   Fm3
....................................uguuugugugCcugguccaug......................................................   1        1   Fm3
....................................uguuugugugucugguccaugu.....................................................   2        0   Fm3
.....................................................................augacuagauccacaCucau......................   26       1   Fm3
.....................................................................augacuagauccacaCucauc.....................   4        1   Fm3
.....................................................................augacuagauccacaCucaucc....................   40       1   Fm3
......................................................................ugacuagauccacaCuca.......................   583      1   Fm3
......................................................................ugacuagauccacaGucau......................   3        1   Fm3
......................................................................ugacuagauccacaCucau......................   38247    1   Fm3
......................................................................ugaAuagauccacauucau......................   1        1   Fm3
......................................................................ugacuagauccacauucau......................   5        0   Fm3
......................................................................ugacuagauccacaAucau......................   1        1   Fm3
......................................................................ugacuagauccacaCucauc.....................   7777     1   Fm3
......................................................................ugacuagauccacauucauc.....................   1        0   Fm3
......................................................................ugacuagauccacaCucaucc....................   109454   1   Fm3
......................................................................ugacuagauccacauucaucc....................   18       0   Fm3
......................................................................ugacuagauccacaGucaucc....................   10       1   Fm3
......................................................................ugacuagauccacaAucaucc....................   6        1   Fm3
......................................................................ugacuagauccacaCucauccu...................   6735     1   Fm3
......................................................................ugacuagauccacauucauccu...................   4        0   Fm3
......................................................................ugacuagauccacaCucauccug..................   5        1   Fm3
.......................................................................gacuagauccacaCucau......................   38       1   Fm3
.......................................................................gacuagauccacaCucauc.....................   6        1   Fm3
.......................................................................gacuagauccacaCucaucc....................   118      1   Fm3
.......................................................................gacuagauccacauucaucc....................   1        0   Fm3
.......................................................................gacuagauccacaCucauccu...................   6        1   Fm3
........................................................................acuagauccacaCucaucc....................   5        1   Fm3

...................................auguuugugugucugguc..........................................................   1        0   Mm2
...................................Uuguuugugugucugguccaug......................................................   1        1   Mm2
...................................auguuugugugucugguccaug......................................................   40       0   Mm2
...................................auguuugugugucugguccaugu.....................................................   1        0   Mm2
.....................................................................augacuagauccacaCucau......................   1        1   Mm2
.....................................................................augacuagauccacaCucaucc....................   6        1   Mm2
......................................................................ugacuagauccacaCuca.......................   48       1   Mm2
......................................................................ugacuagauccacaCucau......................   2319     1   Mm2
......................................................................ugacuagauccacaCucauc.....................   765      1   Mm2
......................................................................ugacuagauccacauucauc.....................   1        0   Mm2
......................................................................ugacuagauccacaAucaucc....................   1        1   Mm2
......................................................................ugacuagauccacaGucaucc....................   1        1   Mm2
......................................................................ugacuagauccacaCucaucc....................   12824    1   Mm2
......................................................................ugacuagauccacauucaucc....................   3        0   Mm2
......................................................................ugacuagauccacaCucauccu...................   591      1   Mm2
......................................................................ugacuagauccacaCucauccug..................   1        1   Mm2
.......................................................................gacuagauccacaCucaucc....................   9        1   Mm2

...................................Guguuugugugucugguccaug......................................................   1        1   Mm1
...................................auguuuguguCucugguccaug......................................................   1        1   Mm1
...................................auguuugugugucugguccaug......................................................   134      0   Mm1
...................................auguuugugugucuggAccaug......................................................   1        1   Mm1
...................................auguuugugugucugguUcaug......................................................   1        1   Mm1
...................................auguuugugugucugguAcaug......................................................   1        1   Mm1
...................................auguuugugugucugguccauguuguaaaagcuca.........................................   1        0   Mm1
.....................................................................augacuagauccacaCucauc.....................   20       1   Mm1
.....................................................................augacuagauccacaCucaucc....................   10       1   Mm1
......................................................................ugacuagauccacauuca.......................   1        0   Mm1
```

```
                                   Star                              Mature
cauucaacauugacagugcagugccagucuuuaggauguuugugugucugguccauguuguaaaagcucaugacuagauccacauucauccugacggcuuggaaucaucua
......................................................................ugacuagauccacaCuca.......................   1309    1   Mm1
......................................................................ugacuagauccacaCucau......................   1692    1   Mm1
......................................................................ugacuagauccacauucauc.....................   1       0   Mm1
......................................................................ugacuagauccacaCucauc.....................   13643   1   Mm1
......................................................................ugacuagauccacauucaucc....................   2       0   Mm1
......................................................................ugacuagauccacaCucaucc....................   26840   1   Mm1
......................................................................ugacuagauccacaGucaucc....................   1       1   Mm1
......................................................................ugacuagauccacaCucauccu...................   1161    1   Mm1
.......................................................................gacuagauccacaCucauc.....................   14      1   Mm1
.......................................................................gacuagauccacaCucaucc....................   14      1   Mm1

...................................Uuguuugugugucugguccaug......................................................   2       1   Fm2
...................................auguuuguguCucugguccaug......................................................   1       1   Fm2
...................................auguuugugugucuggGccaug......................................................   1       1   Fm2
...................................auguuugugugucugguccaug......................................................   403     0   Fm2
...................................auguuugCgugucugguccaug......................................................   1       1   Fm2
...................................auguuugugugucuggucUaug......................................................   1       1   Fm2
...................................auguuuguguAucugguccaug......................................................   1       1   Fm2
...................................auguuugugugucugguccaGg......................................................   4       1   Fm2
...................................auguuugugugucugguAcaug......................................................   1       1   Fm2
...................................auguuugugugucuggCccaug......................................................   1       1   Fm2
...................................auguuugugugucugguGcaug......................................................   2       1   Fm2
...................................auguuugugugucugguUcaug......................................................   2       1   Fm2
...................................aCguuugugugucugguccaug......................................................   1       1   Fm2
...................................auguuugugugucugguccaugA.....................................................   1       1   Fm2
......................................................................ugacuagauccacaCuca.......................   30      1   Fm2
......................................................................ugacuagauccacaCucau......................   312     1   Fm2
......................................................................ugacuagauccacauucauc.....................   9       0   Fm2
......................................................................ugacuagauccacaCucauc.....................   4299    1   Fm2
......................................................................ugacuagauccacaAucaucc....................   2       1   Fm2
......................................................................ugacuagauccacaCucaucc....................   17744   1   Fm2
......................................................................ugacuagauccacauucaucc....................   3       0   Fm2
......................................................................Cgacuagauccacauucaucc....................   1       1   Fm2
......................................................................uUacuagauccacauucaucc....................   1       1   Fm2
......................................................................ugacuagauccacaCucauccu...................   681     1   Fm2
......................................................................ugacuagauccacaCucauccug..................   5       1   Fm2
.......................................................................gacuagauccacaCucaucc....................   4       1   Fm2
........................................................................acuagauccacaCucauc.....................   1       1   Fm2

...................................auguuugugugucugguc..........................................................   1       0   Fm1
...................................auguuugugugucugguccaGg......................................................   1       1   Fm1
...................................auAuuugugugucugguccaug......................................................   1       1   Fm1
...................................auguuugugugucuggCccaug......................................................   1       1   Fm1
...................................auguuugugugucuggGccaug......................................................   2       1   Fm1
...................................auguuugugugucugguUcaug......................................................   1       1   Fm1
...................................auguuugugugucuggucUaug......................................................   1       1   Fm1
...................................auguuugugugucugguGcaug......................................................   1       1   Fm1
...................................auguuugugugucugguAcaug......................................................   2       1   Fm1
...................................auguuugugugucugguccaug......................................................   219     0   Fm1
...................................auguuugugugucugguccaugA.....................................................   1       1   Fm1
...................................auguuugugugucugguccauguuguaGaagcuca.........................................   3       1   Fm1
.....................................................................augacuagauccacaCucauc.....................   10      1   Fm1
.....................................................................augacuagauccacaCucaucc....................   3       1   Fm1
......................................................................ugacuagauccacaCuca.......................   104     1   Fm1
......................................................................ugacuagauccacaCucau......................   1663    1   Fm1
......................................................................ugacuagauccacaCucauc.....................   2197    1   Fm1
......................................................................ugacuagauccacaCucaucc....................   13070   1   Fm1
......................................................................ugacuagauccacauucaucc....................   8       0   Fm1
......................................................................ugacuagauccacaCucauccu...................   613     1   Fm1
......................................................................ugacuagauccacaCucauccug..................   1       1   Fm1
.......................................................................gacuagauccacaCucauc.....................   1       1   Fm1
.......................................................................gacuagauccacaCucaucc....................   5       1   Fm1
.......................................................................gacuagauccacaCucauccu...................   1       1   Fm1
```
